# Optimization of Ring Laser Gyroscope Bias Compensation Algorithm in Variable Temperature Environment

**DOI:** 10.3390/s20020377

**Published:** 2020-01-09

**Authors:** Jun Weng, Xiaoyun Bian, Ke Kou, Tianhong Lian

**Affiliations:** Department of Precision Instruments, school of Mechanical and Instrumental Engineering, Xi’an University of Technology, Xi’an 710048, Shaanxi, China; 18182422360@163.com (X.B.); kouke@xaut.edu.cn (K.K.); thlian@xaut.edu.cn (T.L.)

**Keywords:** ring laser gyroscope, temperature compensation, piecewise least squares fitting, overlap

## Abstract

In a high accuracy strapdown inertial navigation system (SINS), the ring laser gyroscope’s (RLG) bias changes and the performance decreases due to factors in the RLG’s self-heating and changes in ambient temperature. Therefore, it is important to study the bias temperature drift characteristics of RLGs in high, low, and variable temperature environments. In this paper, a composite temperature calibration scheme is proposed. The composite temperature model introduces the derivative term and the temperature derivative cross-multiplier on the basis of the static model and sets the overlap regions for the piecewise least squares fitting. The results show that the composite temperature model can compensate the bias trend term well at ambient temperature, improve the fitting accuracy, and smooth the output curve. The compensation method has a small amount of calculations and flexible parameter design. The precision of the laser gyros in one SINS is improved by about 64.9%, 15.7%, and 3.6%, respectively, which has certain engineering application value.

## 1. Introduction

As the ideal device for strapdown inertial navigation systems (SINSs), the ring laser gyroscope (RLG) has the characteristics of a short starting time, large dynamic range, high reliability, a long life, and digital output. Its performance plays a decisive role in the accuracy of the SINS [1,2,3]. In addition to the performance of the manufacturing process and internal structure, the gyro accuracy is also related to the test environment and working conditions. SINSs [4] are applied in many systems such as those for missiles, rockets, ships, and ground vehicles. The temperature of the working environment of the system can vary greatly, which requires the variable temperature characteristics found in RLGs. In the engineering application process, the inherent temperature characteristics of the RLG means the gyro bias changes along with temperature, which restricts further improvement of the gyro performance. Therefore, it is necessary to study the effect of temperature characteristics on the bias and the real-time compensation algorithm.

Many researchers have studied the bias compensation of the RLG using different compensation models and algorithms to provide an accurate and stable output. Hong and Lee [5] proposed a link between the offset drift compensation of the RLG and the external environment, especially the thermal effect. They found that the deviation drift is linearly proportional to the temperature and temperature gradients, which removes thermal effects from the gyroscope, and that the rate of change in temperature and the temperature gradient have the same effect on the gyro. Zhang [6,7] studied the bias and multi-temperature point, temperature, temperature gradient, and temperature rate of the machine gyro, and obtained a temperature compensation model by the stepwise regression method. The temperature characteristics of the mechanically dithered RLG were also studied by temperature experiments. The radial basis neural network (RBFNN) was used for temperature drift identification, whose parameter identification was performed by the orthogonal least squares (OLS) method. Zhao and Li [8] established a bias temperature compensation model based on a temperature quadratic term and temperature gradient. Ouyang and Chen [9] selected multiple possible environment terms as bias compensation state variables, then used the stepwise regression analysis method to select the significant terms. They established a precise mathematical physics model for the temperature compensation of an RLG. Ge and Chen [10] proposed a new three-axis RLG temperature error model, and a test method based on the principle of least squares. Tao and Tang [11] proposed a method to establish an initial model by the three-time spline interpolation method and iteratively calculated the model deviation correction spline curve. They entered the temperature error model of a laser gyro and a quartz flexible accelerometer into a digital signal processor (DSP), and the real-time compensation of the inertial output was finally realized by the navigation computer. He and Tao [12] analyzed the correlations between bias and temperature, temperature change rate and temperature gradient, and established a second-order dynamic temperature model with multiple temperature points in a complex temperature changing environment, optimizing the contribution to the maximum gyro bias. Li and Zhang [13] investigated the effect of variation of light intensity and dither frequency on RLG drift, then used the support vector machine algorithm to establish a data fusion model that was then used to process the original data in order for them to obtain more accurate results. Geng and Fei [14] proposed an RLG bias temperature compensation method, which used an output signal combined with a least squares support vector machine (LS-SVM) algorithm. Guo and Geng [15] proposed a new scheme for the temperature compensation of the RLG bias system based on LS-SVM. Ding and Zhang [16] proposed an improved RBFNN, based on the Kohonen network and the OLS algorithm, to overcome the effect of temperature on the RLG bias. The improved method combines the pattern classification ability of the Kohonen network with the optimal selection ability of the OLS algorithm, avoiding the random selection of the RBFNN center, and improving the compensation precision of the RBFNN. Cheng and Fang [17] proposed a multi-temperature variable input modeling method based on particle swarm optimization (PSO)-adjusted SVM. First, temperature drift data for modeling are preprocessed by an adaptive forward linear prediction (FLP) filter. Then, the SVM method is used to construct the drift model to ensure generalization ability. The PSO algorithm is used to adjust the parameters of the SVM and improve the accuracy in building the model. Wang and Niu [18] studied the influence mechanisms of temperature, temperature rate, and temperature gradient on inertial devices. Stepwise regression analysis and back propagation (BP) neural network were utilized to identify the parameters of temperature error models. Moreover, the effectiveness of the two methods was proved by temperature error compensation tests. Tao and Li [19] analyzed the effect of high frequency oscillator voltage (UHFO) on the total reflection prisms laser gyro (TRPLG) bias. Moreover, a compensation method based on iteratively re-weighted least squares support vector machines (IR-LSSVM) was proposed, which can improve the TRPLG bias stability effectively.

For the above research results, the influence of temperature on the gyro bias is mainly attributed to the influences of the temperature, temperature gradient, and temperature rate. The compensation algorithms mainly adopt the least squares (LS), spline interpolation, stepwise regression, RBFNN, and SVM methods. Compared with LS, stepwise regression, RBFNN, and SVM are suitable for non-linear fitting, but the amount of calculation is large. Cubic spline interpolation has second-order smooth continuity for nonlinear fitting, but third-order interpolation is required for all temperature ranges. The least squares method is most widely used in the temperature-modeling of inertial devices. However, the least squares method has certain limitations, among which is the fact that polynomial fitting can only achieve higher fitting accuracy under the condition that the sample curve is monotonous without obvious fluctuation, and the fact that piecewise fitting can hardly guarantee smooth continuity at the segment points. Therefore, considering the calculation amount, parameter design, and economy in engineering application, a piecewise least squares fitting method with overlaps is proposed to guarantee smooth continuity at the segment points. Moreover, for the second-order model, the second order of the non-overlaps and the third order of the overlaps can be used to ensure the smooth continuity of the segmented points. In addition, in order to improve the stability and accuracy of the RLG in a dynamic environment, the temperature derivative and the temperature derivative cross-multiply dynamic error terms are introduced, and their parameters are identified based on the static temperature compensation. 

## 2. Formation Mechanism of RLG Bias Temperature Drift Error

RLG bias is caused by any non-reciprocal effect of clockwise and counterclockwise traveling waves present in the cavity, such as the Langmuir flow effect of the activation medium, the flow effect of the inactive medium, the bias caused by the magnetic field, poor loss, an unstable blocking threshold, an unequal threshold of cis-anti-blocking, multi-mode coupling effect, and zero drift. They all produce the same effect as the angular velocity of rotation, which results in a frequency difference in the reverse traveling wave. The frequency difference caused by these effects will eventually be superimposed on the frequency difference corresponding to the rotation and output from the optical signal. This interferes with the measurement of the rotation. If the RLG bias is stable and repeatable, it can be compensated in real time by a computer. However, in fact, the bias is inherently variable and has some random nature, which is difficult to control. So, bias is an important source of error for the RLG [20,21,22].

The effect of temperature on the gyro bias is significant [23,24,25,26,27,28,29]: (1) Regarding the heat source, when the gyro is working the source needs to heat up, and it takes several hours to reach equilibrium; when conditions such as ambient temperature change, the temperature field will become more complicated and more difficult to balance, which will affect the performance of the gyro. (2) In terms of physical properties, the refractive index of the gas, the thermal conductivity of the material, and the optical properties of the optical device also change along with the temperature. (3) Regarding the geometrical characteristics, the thermal expansion and contraction and bending deformation of the device can cause the optical path to change and the loss of the resonant system to increase. (4) Finally, a change in the temperature field causes the flow field to change, which causes the discharge currents of the two arms to be unbalanced and aggravates the Langmuir flow effect. These changes will affect the RLG bias. Temperature affects almost all factors such as physical parameters, geometric deformation, gas flow field, and so on.

## 3. RLG Temperature Compensation Model and Algorithm

The RLG bias temperature compensation requires fitting the experimental data to obtain the trend curve of bias with temperature. However, the trend curves obtained by different curve fitting methods are different in their fitting accuracy.

Due to the high accuracy of least squares polynomial fitting, it has a wide range of applications. For many data and complex curves, the results are not ideal when the low-order least squares polynomial fitting is used. When the high-order least squares polynomial fitting is used, the fitting accuracy and reliability are reduced. Therefore, an RLG output pulse curve fitted by using a piecewise least squares polynomial is proposed in this paper.

### 3.1. Determination of Segments

The RLG output pulses curve always has inflection points or abrupt data points. For the gyro output curve fitting function y(x), the deviation is large at the valley peak of the curve when the output pulses curve is fitted using a low-order least squares fit. So, the extreme point of the fitted curve should be limited to a certain range. That is to say, if the highest order of the data is *n*, let the first derivative of the fitted curve $y^{\prime }\left(x\right)$ be zero and the second derivative $y^{\prime \prime }\left(x\right)$ not be zero. Then, the extreme point of the curve can be obtained, that is $x_{0}^{\prime },x_{1}^{\prime },\cdots ,x_{i}^{\prime }\left(i\leq n-1\right)$, and $0<x_{0}^{\prime }<x_{1}^{\prime }<\cdots <x_{i}^{\prime }<x_{\max}$. 

The segment point should be set at the inflection point of the curve. For the gyro output y(x), let the $y^{\prime \prime }\left(x\right)$ be zero and its third derivative $y^{\prime \prime \prime }\left(x\right)$ not be zero. Therefore, the inflection points of the curve can be obtained, that is $x_{0}^{\prime \prime },x_{1}^{\prime \prime },\cdots ,x_{k}^{\prime \prime }\left(k\leq n-2\right)$, and $0<x_{0}^{\prime \prime }<x_{1}^{\prime \prime }<\cdots <x_{k}^{\prime \prime }<x_{\max}$. The inflection points of the curve are the segment points.

### 3.2. Overlap

For complex curve fitting, the curve may deviate to some extent. Therefore, the inflection points of the fitted curve may not be the inflection points of the output curve. When setting the segmentation points, the selection of a segmentation point should be within a certain neighborhood of the calculated inflection point, namely, the abscissas of the segmentation points are $x_{k}\in \left(x_{k}^{\prime \prime }-\Delta ,x_{k}^{\prime \prime }+\Delta \right)$ and k≤n−2, and Δ represents the neighborhood of the calculated inflection point, namely, half of the overlap.

Because the offset of the inflection points of the fitted curve is unknown, it is not possible to give an accurate value for the neighborhood of the inflection point. To ensure the curve is smooth, the segmentation point should be set near the inflection point and between the left and right extreme values at the inflection point. The abscissa of the *k*-th segment point should satisfy $x_{k}^{\prime }<x_{k}^{\prime \prime }-\Delta \leq x_{k}\leq x_{k}^{\prime \prime }+\Delta <x_{k+1}^{\prime }$ and k≤n−2.

In practical applications, because the segment points are set by the inflections, the number of segments can be divided according to the unevenness of the output pulse curve. In order to make the curve fit smooth, an overlap is set in the neighborhood of the segment point. In addition, the range of the overlap is smaller than each segment.

### 3.3. Multi-Segment Continuous Least Squares

It is assumed that $x_{i}$ is the gyro temperature, and $y_{i}$ is the gyro raw output pulse. Considering to divide the data into *K* segments, the data set of each segment can be expressed as

(1) $$\begin{array}{c} S_{k}=\left\{\left(x_{k,i},y_{k,i}\right)\right\},\;i=1,2,\cdots ,n_{k}\;,\;k=1,2,\cdots ,K\\ \mathrm{subject}\;\mathrm{to}\;\left\{ \begin{array}{l} n_{1}+n_{2}+\cdots +n_{k}=n\\ x_{k-1,n_{k-1}}<x_{k,i}^{}<x_{k+1,1},1\leq i\leq n_{k} \end{array}\right. \end{array}$$

where $n_{k}$ represents the number of data in the *k*-th segment, and $x_{k,i}^{}$ represents that there are $n_{k}$ data in the *k*-th segment, namely, the temperature value in the *k*-th segment. 

The fitting function y(x) can be determined as:

(2) $$y(x)=\left\{ \begin{array}{c} y_{1}\left(x\right)=\sum_{j=1}^{m_{1}}\alpha_{1,j}^{}h_{1,j}^{}\left(x\right)=X_{1}\alpha_{1},x\leq x_{1,n_{1}}\\ y_{2}\left(x\right)=\sum_{j=1}^{m_{2}}\alpha_{2,j}^{}h_{2,j}^{}\left(x\right)=X_{2}\alpha_{2},x_{1}<x\leq x_{2,n_{2}}\\ \;\vdots \\ y_{K}\left(x\right)=\sum_{j=1}^{m_{K}}\alpha_{K,j}^{}h_{K,j}^{}\left(x\right)=X_{K}\alpha_{K},x>x_{K-1,n_{K-1}} \end{array}\right.$$

where $\left\{h_{k,j}^{}\left(x\right)\right\},j=1,2,\cdots ,m_{k}$ is a set of the linearly independent basis functions in the segment of $S_{k}$, $m_{k}$ represents the number of basis functions in the segment of $S_{k}$, and $\left\{\alpha_{k,j}^{}\right\},j=1,2,\cdots ,m_{k}$ is a set of fitting coefficients in the segment of $S_{k}$. In this paper, $\left\{h_{k,j}^{}\left(x\right)\right\}$ represents a set of the temperature-dependent terms. Let $y={\left[y_{1}^{T},y_{2}^{T},\cdots ,y_{K}^{T}\right]}^{T}$, $\alpha ={\left[\alpha_{1}^{T},\alpha_{2}^{T},\cdots ,\alpha_{K}^{T}\right]}^{T}$, and $X=\left[\begin{array}{cccc} X_{1} & 0 & \cdots & 0\\ 0 & X_{2} & \cdots & 0\\ \vdots & \vdots & & \vdots \\ 0 & 0 & \cdots & X_{K} \end{array}\right]$.

All the fitting coefficients can be obtained by the least squares method:

(3) $$\alpha ={\left(X^{T}X\right)}^{-1}X^{T}y$$

Considering that X is a diagonal matrix, the inversion operation in Equation (3) can be simplified as the inversion of the block matrix on the diagonal, which means

(4) $$\left\{ \begin{array}{l} \alpha_{1}={\left(X_{1}^{T}X_{1}^{}\right)}^{-1}X_{1}^{T}y_{1}^{}\\ \alpha_{2}={\left(X_{2}^{T}X_{2}^{}\right)}^{-1}X_{2}^{T}y_{2}^{}\\ \;\vdots \\ \alpha_{K}={\left(X_{K}^{T}X_{K}^{}\right)}^{-1}X_{K}^{T}y_{K}^{} \end{array}\right.$$

Taking the overlap in the neighborhood of the *k-th* segment point $x_{k}$ as an example, the overlap is $\left(x_{k}-\Delta )<x\leq (x_{k}+\Delta \right)$. The following processing is required for compensation calculation:

(5) $${\hat{y}}_{k}=\kappa_{1}\sum_{j=1}^{m_{k}}\alpha_{k,j}^{}h_{k,j}^{}\left(x\right)+\kappa_{2}\sum_{j=1}^{m_{k+1}}\alpha_{k,j+1}^{}h_{k+1,j}^{}\left(x\right)$$

**Figure 1:**
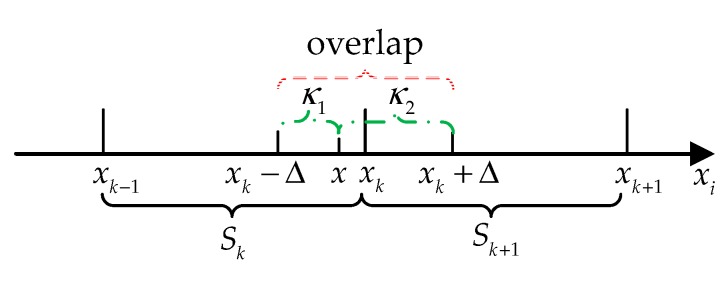
Schematic diagram of the overlap calculation.

Among them, the weighting coefficients are $\kappa_{1}=(x_{k}-x+\Delta )/2\Delta ,\;\kappa_{2}=1-\kappa_{1}$. This ensures the continuity of the least squares fit of the two consecutive segments in the overlap. Figure 1 shows the schematic diagram of the overlap calculation. 

In Figure 1, the abscissa represents the temperature $x_{i}$, the dotted line is the overlap, and the dash-dotted lines are the weight of each part in the overlap. Further, suppose that the piecewise function uses a quadratic curve, namely, $\left\{h_{k,j}^{}\left(x\right)\right\}=\left\{1,x,x^{2}\right\}$, and substitute $\kappa_{1},\;\kappa_{2}$ into Equation (5)

(6) $$\begin{array}{cc} {\hat{y}}_{k} & =\frac{\left(x_{k}-x+\Delta \right)}{2\Delta }\sum_{p=0}^{2}\alpha_{k}(p)x_{}^{p}+\frac{\left(x-x_{k}+\Delta \right)}{2\Delta }\sum_{p=0}^{2}\alpha_{k+1}(p)x_{}^{p}\\ & =\frac{1}{2\Delta }\sum_{p=0}^{2}\left[\alpha_{k+1}(p)-\alpha_{k}(p)\right]x_{}^{p+1}-\frac{1}{2\Delta }\sum_{p=0}^{2}x_{k}\left[\alpha_{k+1}(p)-\alpha_{k}(p)\right]x_{}^{p}+\frac{1}{2}\sum_{p=0}^{2}\left[a_{k}(p)+a_{k+1}(p)\right]x_{}^{p}\\ & =\frac{1}{2\Delta }\left\{\sum_{p=0}^{2}\left[\alpha_{k+1}(p)-\alpha_{k}(p)\right]x_{}^{p+1}-\sum_{p=0}^{2}x_{k}\left[\alpha_{k+1}(p)-\alpha_{k}(p)\right]x_{}^{p}+\sum_{p=0}^{2}\Delta \left[\alpha_{k}(p)+\alpha_{i+1}(p)\right]x_{}^{p}\right\} \end{array}$$

where p represents the order of the basis function in a piecewise function. 

It can be seen that after the weighting process, the compensation function of the overlap essentially changes from the original quadratic curve to the cubic curve. In theory, the higher-order fitting function has stronger nonlinearity. Therefore, only performing high-order curve fitting on overlaps can not only ensure the continuity of the segmented points but also reduce the fitting-order of non-overlaps. It avoids the problem of low boundary fitting accuracy caused by directly using the high-order model to fit the whole temperature range. It can be seen that each segment can use a different basis function, and the maximum power of the fitted curve in the overlap is one more than the power of the non-overlap. Thus, this algorithm is flexible and is especially suitable for an RLG with a long test time. When the data of a certain temperature range are lost or incorrect, only the experiment of that temperature range can be done, which can help us save time and reduce workload.

## 4. Temperature Experiment and Results Analysis

### 4.1. Temperature Test System

**Figure 2:**
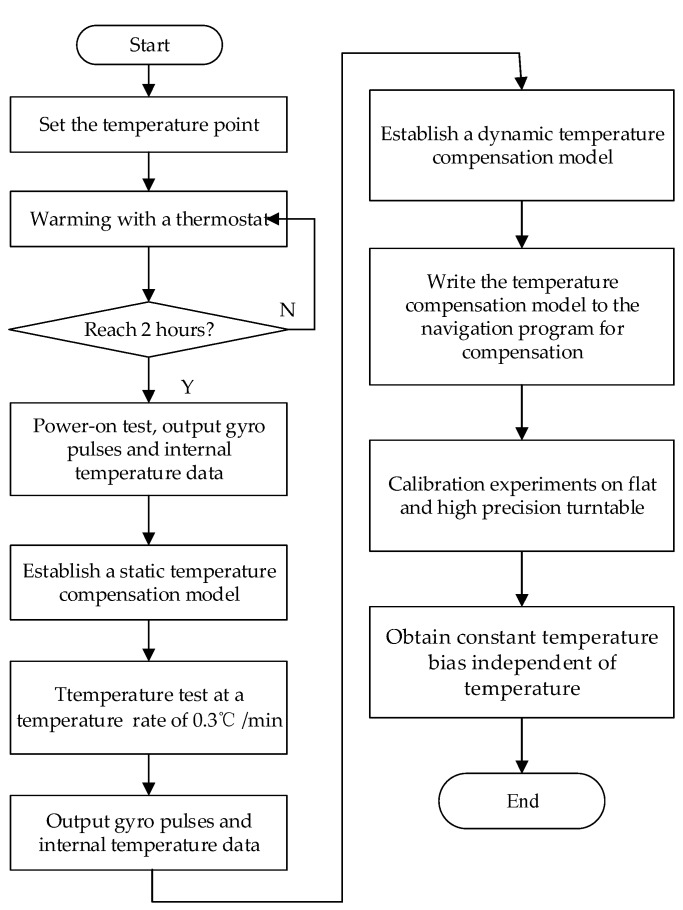
The ring laser gyroscope (RLG) temperature calibration flow chart.

**Figure 3:**
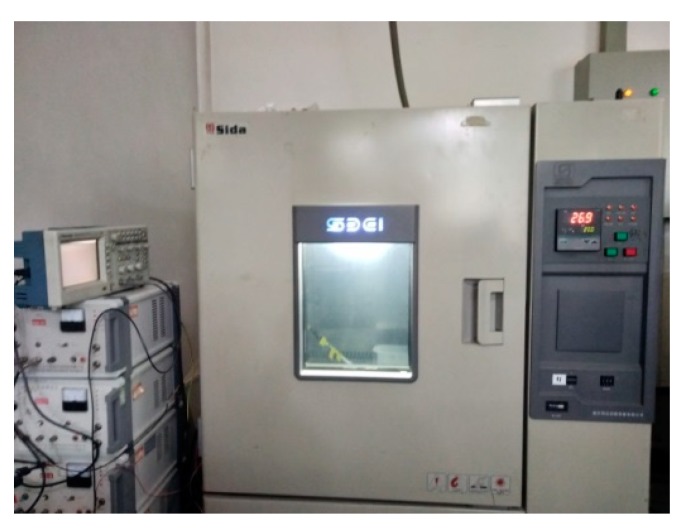
The thermostat for RLG temperature experiments.

The temperature test of the RLG usually requires static and dynamic tests. The static and dynamic conditions here are defined by whether the ambient temperature changes. The static test focuses on analyzing the characteristics of the gyro at different temperature points, while the dynamic test can better investigate the influence of temperature shock on the system performance during the rising and cooling processes in a wide temperature range. By referring to the practical application background of this system, a method combining dynamic and static temperature modeling was designed to effectively identify model parameters. During the experiment, a medium-precision INS consists of three RLGs, of which the *X*-axis, *Y*-axis, and *Z*-axis correspond to the local east, north, and up directions, respectively. The RLG temperature calibration flow chart is shown in Figure 2, and the thermostat for the RLG temperature experiment is shown in Figure 3.

### 4.2. Overlap Analysis

**Figure 4:**
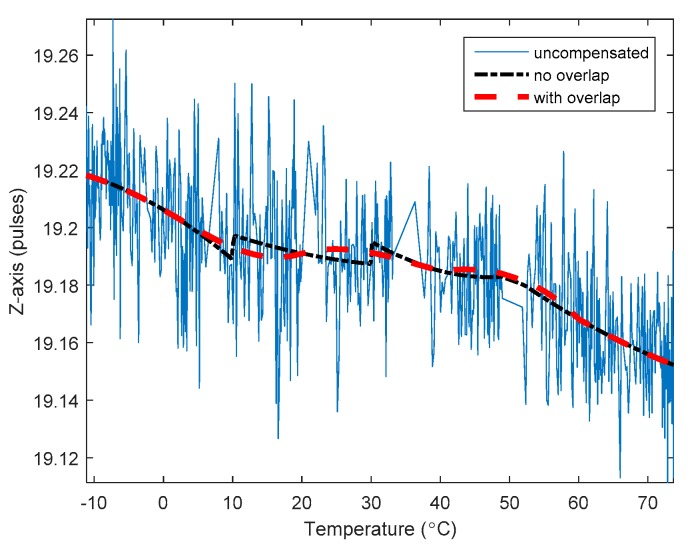
Static output value of the Z-axis RLG and its fitted curve—with and without overlap.

The RLG static test data are used to calculate the temperature parameters. The curve is fitted with overlap and without overlap. Take the *Z*-axis gyroscope as an example to illustrate the improvement in the nonlinearity of the fitted curve produced by the overlaps. Figure 4 shows the static output value of the *Z*-axis RLG and its fitted curve—with and without overlap. The blue solid line represents the original output pulses, and the black stippled line represents the RLGs fitted curve after the piecewise least squares compensation without overlap. It can be seen that there is a significant discontinuity at the segment points. After the overlap is used in the piecewise least squares compensation, the fitting curve is smooth. The RLGs bias before and after temperature compensation is shown in Table 1.

It can be seen from the above that using overlaps improves the nonlinearity of the fitting curve and the accuracy of the RLG in the static temperature tests.

### 4.3. Static Temperature Compensation Model and Results Analysis

For the RLG, the constant bias can be compensated by calibration, and the trend bias can only be compensated by establishing the temperature model. Due to the little influence of temperature changing rate and temperature gradient on the constant bias, the static model did not consider it. Therefore, based on the RLG constant value term, temperature linear term and temperature quadratic term, a static temperature compensation model was established and its compensation effect was analyzed. The model is as follows:

(7) $$y_{k}=\alpha_{k,0}+\alpha_{k,1}x+\alpha_{k,2}x^{2}$$

where $y_{k}$ is the number of RLG compensation pulses at the temperature x in the *k*-th segment, and $\alpha_{k,j},(k=1,2,\cdots ,K;j=0,1,2)$ are the piecewise least squares fitting coefficients in the *k*-th segment.

**Figure 5:**
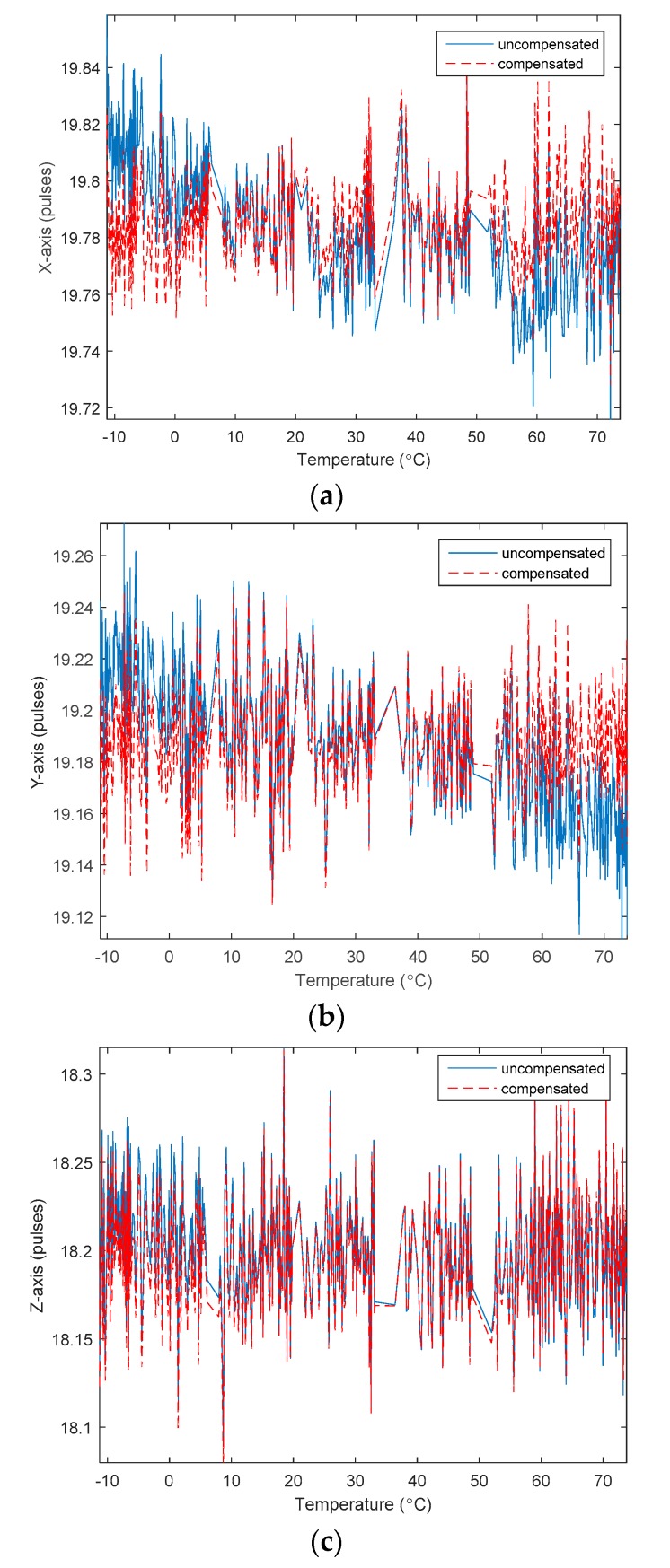
Bias of RLGs before and after the static temperature model compensation. (**a**) *X*-axis of gyro; (**b**) *Y*-axis of gyro; (**c**) *Z*-axis of gyro.

The experimental data of the static temperature points before and after the static temperature model compensation are shown in Figure 5. 

For static test data, considering the stability of the bias curve, the temperature is divided into four segments, and the overlapping interval is 10 °C. The piecewise least squares fitting coefficients of static data are shown in Table 2.

**Figure 6:**
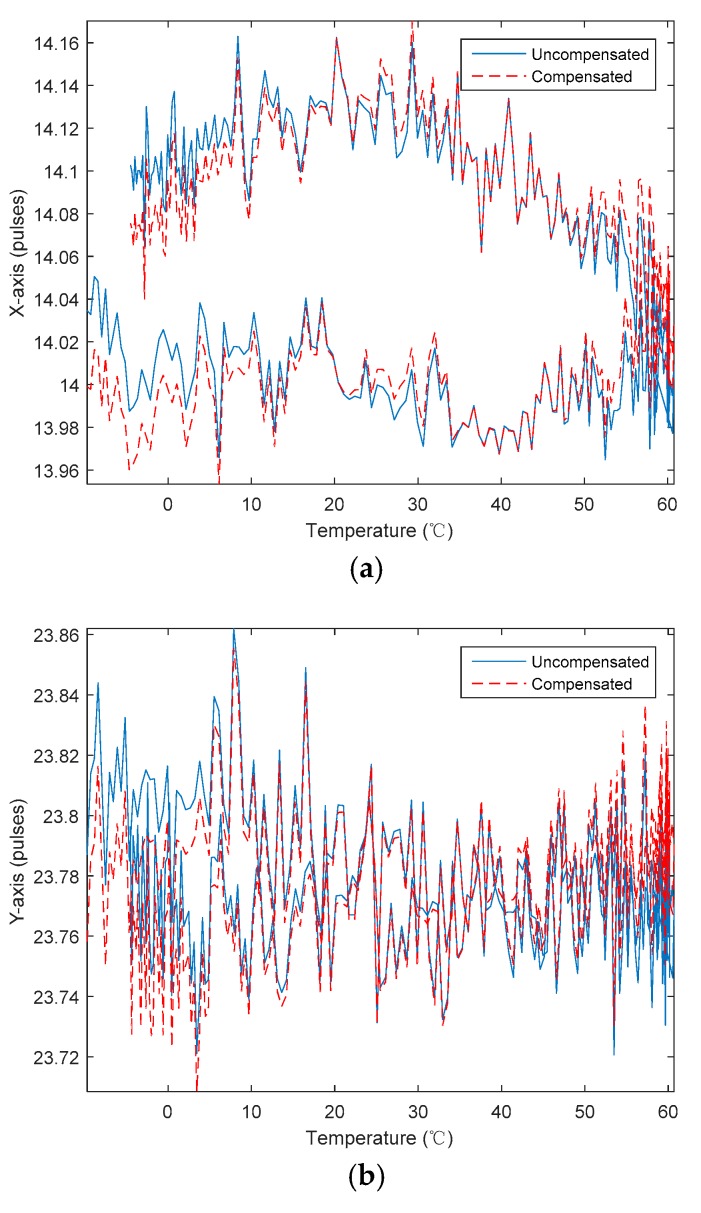

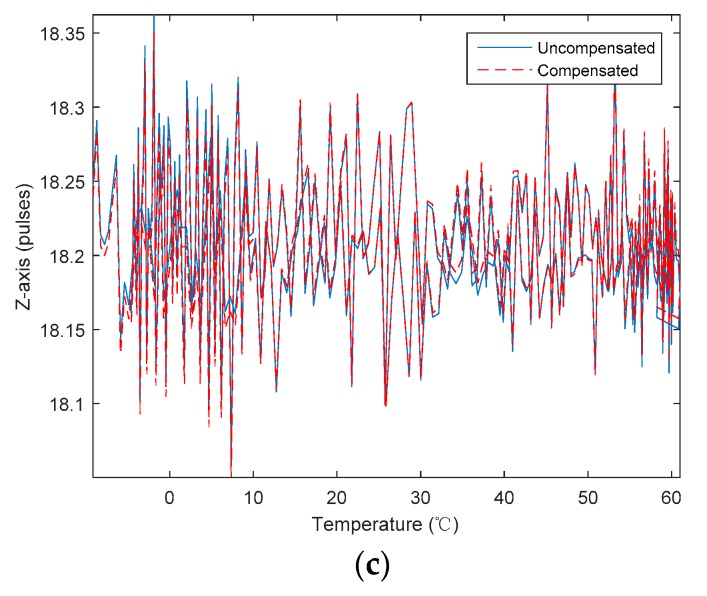
Bias of RLG before and after the static temperature model compensation. (**a**) *X*-axis of gyro; (**b**) *Y*-axis of gyro; (**c**) *Z*-axis of gyro.

The dynamic temperature experimental data of the RLGs were subjected to temperature compensation verification using the static model parameters in Table 2. Figure 6 shows the output curve of each direction of the RLG before and after compensation.

It can be seen that the static temperature compensation model improves the performance of the gyro when the system has a particularly small temperature change rate. However, for complex environments such as those with a large temperature change rate or a large temperature change range, the static temperature model has no significant effect on dynamic temperature compensation, and there is hysteresis in the temperature cycling. Therefore, it is necessary to propose a dynamic model.

### 4.4. Composite Temperature Compensation Model and Results Analysis

**Figure 7:**
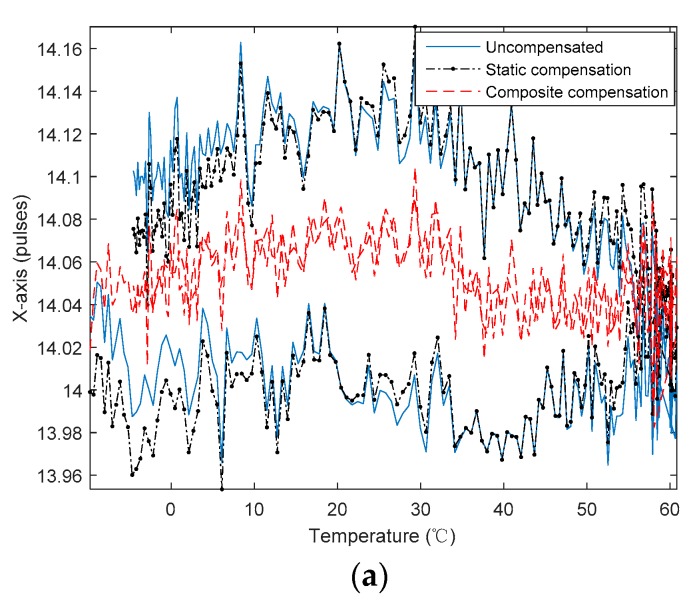

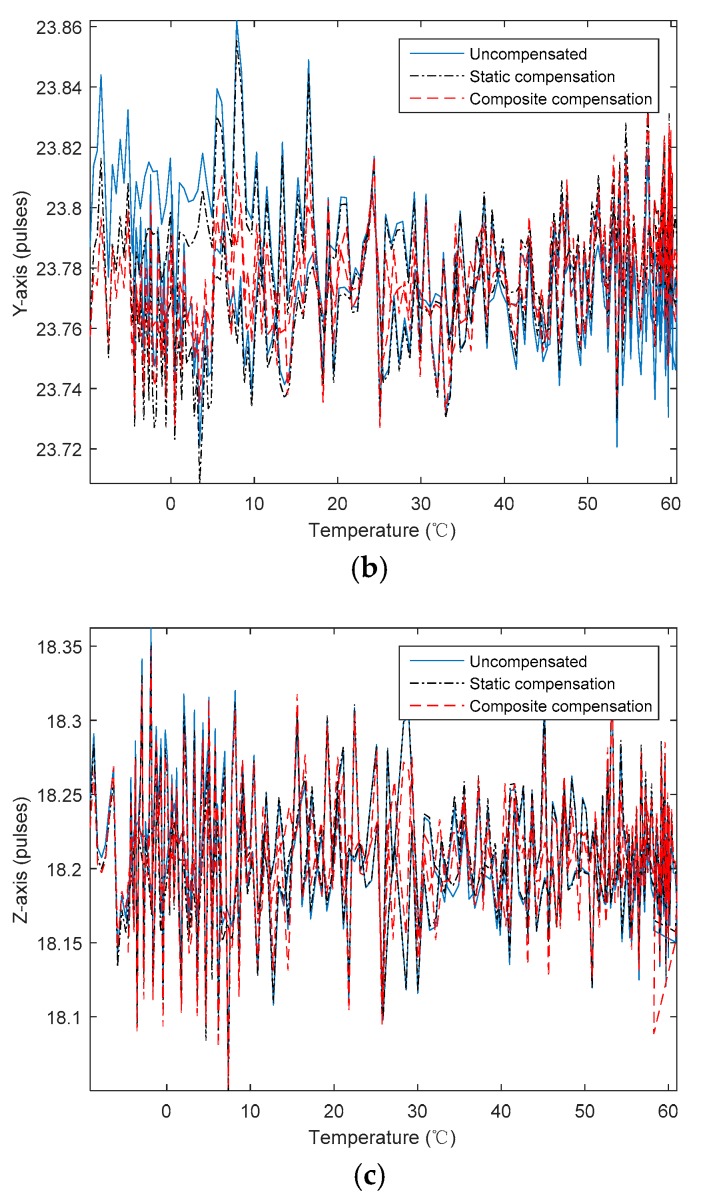
The RLG’s bias curve with static and composite temperature model compensation. (**a**) *X*-axis of gyro; (**b**) *Y*-axis of gyro; (**c**) *Z*-axis of gyro.

In order to reduce the influence of hysteresis on the RLG accuracy in the variable temperature test, the temperature derivative term and the cross-term of temperature and temperature derivative were introduced. Then, their parameters were identified by the piecewise least squares fitting algorithm with the overlaps based on the residuals of the static compensation. Figure 7 shows the RLG’s bias curves with static and composite temperature model compensation.

Static temperature compensation model:

(8) $$y_{k,\mathrm{static}}={\tilde{y}}_{k}-\left(\alpha_{k,0}+\alpha_{k,1}x+\alpha_{k,2}x^{2}\right)$$

Composite temperature compensation model:

(9) $$y_{k,\mathrm{comp}}=y_{k,\mathrm{static}}-\left(\alpha_{k,3}\left(dx/dt\right)+\alpha_{k,4}x\left(dx/dt\right)\right)\;$$

It can be seen from Figure 7 that the hysteresis of the RLG’s *X*-axis was significantly improved. The RLG’s *Y*-axis and *Z*-axis both have a small increase because the original data had low hysteresis. Table 3 shows the RLG’s bias value before and after temperature compensation.

From Table 3, the static compensation of the *X*-, *Y*-, and *Z*-axial RLGs produces little or no improvement to the temperature-changing environment gyro bias. After the composite temperature compensation model, the hysteresis phenomenon is improved, and the bias value is, respectively, reduced by 64.9%, 15.7%, and 3.6%.

## 5. Conclusions

From the comparison of the curve and bias before and after static and composite temperature compensation, using static and dynamic models and algorithm optimization not only reduces the bias value but also eliminates the trend of bias with temperature change. The bias changes with the temperature almost horizontally. That is to say, the output of the RLG is zero at zero input angular rate at various ambient temperatures. At the same time, the degree of bias dispersion has also been improved. This indicates that the cross-term of temperature and temperature rate should be introduced into the RLG bias compensation model to make the error model complete. Additionally, a parameter identification method based on the residual of the static compensation reduces the RLG bias efficiently. On the compensation algorithm of piecewise least squares fitting, using the overlaps method reduces the calculation amount, and makes the models flexible and the fit smooth. Moreover, it fully meets the real-time compensation and economic requirements of the project. At present, this algorithm has been embedded in the DSP for a SINS temperature compensation, and the compensation effect is good. Slightly extended, this piecewise least squares fitting algorithm can be applied to the temperature compensation of the accelerometer, so the model has strong engineering practical value.

## Figures and Tables

**Table 1:** The RLGs bias before and after temperature compensation (pulses).

	Uncompensated	Compensated	Compensated (Overlap)
*X*-axis	0.0233	0.0174	0.0173
*Y*-axis	0.0280	0.0190	0.0189
*Z*-axis	0.0335	0.0086	0.0071

**Table 2:** Table of fitting coefficients of static data.

	Temperature Segment (*k*)	Model Parameters		
		$\alpha_{0}$	$\alpha_{1}$	$\alpha_{2}$ (×10^−5^)
*X*-axis	1/(−15–15 °C)	0.0173	−0.0014	1.7535
	2/(5–35 °C)	0.0064	0.0004	−4.2567
	3/(25–60 °C)	−0.0551	0.0026	−3.302
	4/(50–80 °C)	0.3390	−0.0118	9.6146
*Y*-axis	1/(−15–15 °C)	0.0184	−0.0014	−3.1281
	2/(5–35 °C)	0.0187	−0.0010	1.3435
	3/(25–60 °C)	0.0884	−0.0040	4.1885
	4/(50–80 °C)	0.1204	−0.0033	1.5919
*Z*-axis	1/(−15–15 °C)	0.0167	0.0004	−2.0700
	2/(5–35 °C)	0.0036	−0.0009	3.9374
	3/(25–60 °C)	0.0635	−0.0042	6.5480
	4/(50–80 °C)	−0.0974	0.0032	−2.7658

**Table 3:** The RLG’s bias before and after temperature compensation (pulses).

	Uncompensated	Static Compensation	Composite Compensation
*X*-axis	0.0533	0.0512	0.0187
*Y*-axis	0.0229	0.0232	0.0193
*Z*-axis	0.0471	0.0467	0.0454
